# Clinical Outcomes and Safety of Intralesional Vitamin D3 for Cutaneous Warts: A Retrospective Cohort Study From Sana'a, Yemen

**DOI:** 10.7759/cureus.112281

**Published:** 2026-07-08

**Authors:** Hanan Alkebsi, Nojood D Albaadani, Mutaia Abuarij, Sumaia Almadani

**Affiliations:** 1 Department of Dermatology, Typical Police Hospital, Sana'a, YEM; 2 Department of Dermatology, 21 September University for Medical and Applied Sciences, Sana'a, YEM; 3 Department of Dermatology, Al Sabeen Maternal Hospital, Sana'a, YEM

**Keywords:** human papillomavirus, immunotherapy, injections, intralesional, retrospective studies, treatment outcome, vitamin d, warts, yemen

## Abstract

Background

Cutaneous warts are common lesions caused by human papillomavirus (HPV). Conventional destructive therapies are often painful and associated with high recurrence rates. Intralesional vitamin D3 has emerged as an immunotherapeutic option, though clinical evidence remains inconsistent. This study evaluated the clinical outcomes and safety of intralesional vitamin D3 in a cohort of Yemeni patients.

Methods

This retrospective cohort study evaluated patients treated at two dermatology clinics in Sana'a, Yemen, between January and June 2025. Consecutive patients meeting the inclusion criteria were enrolled. Sixty-one patients with cutaneous warts received intralesional vitamin D3 (60,000-150,000 IU per session) biweekly for up to six sessions. The primary outcome was complete cure, defined as the total disappearance of all treated warts and restoration of normal skin texture. Secondary outcomes included partial response (≥50% reduction), adverse events, and recurrence rates. Data were analyzed using descriptive statistics and univariate logistic regression.

Results

The median patient age was 18 years (IQR 15-34), and 77.0% (n = 47) were female. Complete cure was observed in 36 patients (59.0%), while 20 (32.8%) showed partial improvement, yielding an overall response rate of 91.8% (95% CI 82.4-96.5). Procedural pain was the most common adverse event (75.4%), followed by transient localized erythema or swelling (13.1%). No serious adverse events or scarring occurred. No recurrence was documented during the six months of follow-up. In univariate analysis, younger age was associated with higher odds of complete response (OR 0.87 per one-year increase, 95% CI 0.81-0.93; p < 0.001).

Conclusions

Intralesional vitamin D3 was associated with a complete response in approximately 60% of patients and demonstrated a favorable short-term safety profile in this retrospective cohort. However, given the retrospective uncontrolled design, modest sample size, and limited follow-up, randomized controlled trials are required to establish definitive efficacy and control for spontaneous regression.

## Introduction

Cutaneous warts are benign epidermal proliferations caused by human papillomavirus (HPV). They are common in children, adolescents, and young adults and may result in pain, cosmetic concern, and functional impairment depending on location [1]. Although spontaneous regression may occur, a subset of lesions persists or recurs after treatment [2,3].

Conventional therapies include cryotherapy, electrocautery, laser ablation, topical keratolytics, and surgical destruction [1,3]. These modalities primarily target visible lesions and may be associated with pain, pigmentary changes, scarring, and recurrence, often requiring repeated sessions, particularly in patients with multiple or recalcitrant disease [1,2].

Intralesional immunotherapy has emerged as an alternative approach that enhances cell-mediated immune responses against HPV-infected keratinocytes and may induce clearance of both treated and distant lesions [2,4]. Agents studied include Candida antigen, measles-mumps-rubella (MMR) vaccine, purified protein derivative (PPD), *Mycobacterium indicus pranii* (MIP) vaccine, and vitamin D3 [4,5].

Vitamin D3 has attracted increasing interest as an intralesional immunotherapeutic agent because of its immunomodulatory and antiproliferative properties. It regulates keratinocyte differentiation and enhances innate immune responses via induction of antimicrobial peptides, including cathelicidin and human beta-defensin [4,6]. Vitamin D3 may also enhance local cell-mediated immune responses, thereby providing a biologically plausible mechanism for clearance of HPV-infected keratinocytes [6,7]. However, reported clinical outcomes remain variable, likely reflecting heterogeneity in study design, patient characteristics, wart type, and treatment protocols.

Despite increasing global interest, evidence from Yemen and the wider Middle Eastern region remains limited. This study therefore aimed to evaluate the clinical outcomes, safety profile, recurrence rate, and predictors of response to intralesional vitamin D3 in patients with cutaneous warts treated in two dermatology clinics in Sana’a, Yemen.

## Materials and methods

Study design and setting

This retrospective cohort study was conducted at the dermatology outpatient clinics of the Typical Police Hospital and Al-Sabain Hospital in Sana’a, Yemen. The study period extended from January 1 to June 30, 2025. The aim was to evaluate the clinical outcomes and safety profile of intralesional vitamin D3 in patients with cutaneous warts under real-world clinical conditions. The study was conducted in accordance with the Declaration of Helsinki.

Ethical considerations

Ethical approval was obtained from the Institutional Review Board of the Modern German Hospital, Sana’a, Yemen (IRB reference no. 2025-014). A waiver of informed consent was granted by the IRB due to the retrospective nature of the study, and all data were anonymized prior to analysis to ensure confidentiality.

Participants

**Figure 1:**
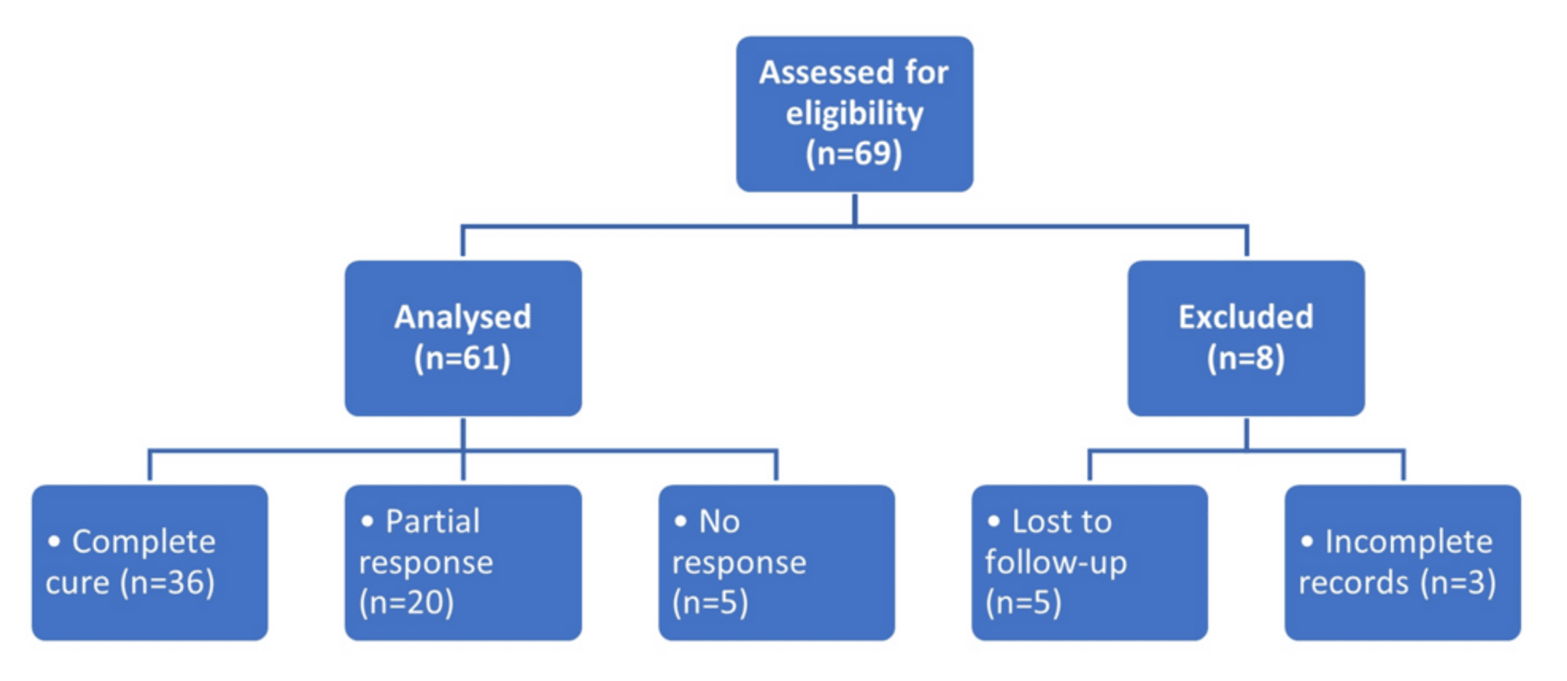
Flow diagram of the patient selection process and clinical outcomes. Source: Primary clinical data collected by the authors.

Of 69 patients screened, 61 consecutive patients meeting the inclusion criteria were included in the final analysis; eight were excluded due to loss to follow-up (n = 5) or incomplete records (n = 3) (Figure 1).

Eligible patients were aged nine years or older with a clinical diagnosis of cutaneous warts, including common, plantar, periungual, and plane types, with persistent lesions lasting several months or longer. Patients were excluded if they were pregnant or breastfeeding, immunocompromised (including HIV infection, organ transplantation, or chronic immunosuppressive therapy), had known hypersensitivity to vitamin D preparations, or presented with genital warts. Both treatment-naïve patients and those with prior unsuccessful therapies were included to reflect routine clinical practice.

Intervention and treatment protocol

Intralesional vitamin D3 (cholecalciferol; Devarol®, Memphis Pharmaceutical Industries, Cairo, Egypt) was administered as an oily solution containing 600,000 IU per 2 mL ampoule (300,000 IU/mL). The injected dose ranged from 60,000 to 150,000 IU per session according to lesion size and clinical judgment, consistent with previously published treatment protocols [8-10].

Intralesional injections were administered biweekly using a 1 mL insulin syringe with a 30-gauge needle after skin disinfection with 70% alcohol. Topical anesthesia (EMLA cream) was used when necessary but not routinely applied. The injected volume ranged from 0.2 to 0.5 mL per session (60,000-150,000 IU), adjusted according to lesion size and clinical judgment.

Only the largest or most symptomatic lesion (index wart) was injected in patients with multiple warts to standardize assessment. In larger lesions, the dose was distributed using a fanning technique. Treatment was discontinued after complete clinical resolution or completion of six treatment sessions.

Outcome measures

The primary outcome was complete clinical response, defined as complete clearance of all baseline warts, including treated and untreated lesions, with restoration of normal skin texture. Secondary outcomes included partial response (≥50% reduction in wart size or number), no response (<50% improvement), overall response (complete plus partial response), adverse events, and recurrence.

Recurrence was defined as the reappearance of lesions at previously cleared sites within six months after complete response. Patients achieving complete response were followed at one, three, and six months post-treatment.

Data collection and definitions

Data were extracted from medical records, including demographic characteristics, wart type, lesion duration, number of lesions, prior treatments, and outcomes. No standardized wart severity scoring system was used, which is acknowledged as a limitation.

Statistical analysis

Data were analyzed using IBM SPSS Statistics for Windows, Version 26.0 (Released 2018; IBM Corp., Armonk, NY, USA). Continuous variables were summarized as median (interquartile range) or mean ± standard deviation, depending on distribution. Categorical variables were presented as frequencies and percentages.

Normality was assessed using the Shapiro-Wilk test. Associations between clinical variables and complete response were evaluated using univariate logistic regression, with odds ratios and 95% confidence intervals reported. Categorical variables were analyzed using chi-square or Fisher’s exact test, and continuous variables using Student’s t test or Welch’s t test where appropriate. A two-sided p-value <0.05 was considered statistically significant.

Multivariable logistic regression was not performed because the sample size and number of outcome events were insufficient to support stable multivariable modeling. Consequently, all inferential analyses should be considered exploratory.

## Results

Baseline characteristics

A total of 61 patients were included in the final analysis. The majority of the cohort was female (77.0%, n = 47). The median age of the cohort was 18 years (IQR: 15-34 years), with the majority of patients (63.9%, n = 39) falling into the 11-20 years age bracket.

Common warts were identified in 31 patients (50.8%), plantar warts in 18 (29.5%), periungual warts in seven (11.5%), plane (flat) warts in one (1.6%), and mixed wart types in four (6.6%). Prior treatment history was present in 30 patients (49.2%), while 31 (50.8%) were treatment-naïve. The mean number of warts was 1.92 ± 1.08, the mean lesion surface area was 22.61 ± 26.62 mm², and the mean disease duration was 5.65 ± 4.54 months. Baseline characteristics are summarized in Table 1.

**Table 1:** Baseline demographic and clinical characteristics of the study cohort (N = 61). Legend: Summary of patient demographics and clinical profiles at baseline. Abbreviations: IQR, interquartile range; SD, standard deviation; mm², square millimeters.

Characteristic	Value
Sex, n (%)	
Female	47 (77.0)
Male	14 (23.0)
Age, years	
Median (IQR)	18 (15-34)
Mean ± SD	23.18 ± 12.44
Age group, n (%)	
11-20 years	39 (63.9)
21-30 years	6 (9.8)
31-40 years	6 (9.8)
41-50 years	8 (13.1)
≥51 years	2 (3.3)
Wart type, n (%)	
Common	31 (50.8)
Plantar	18 (29.5)
Periungual	7 (11.5)
Plane	1 (1.6)
Mixed	4 (6.6)
Wart characteristics	
Number of warts (mean ± SD)	1.92 ± 1.08
Surface area, mm² (mean ± SD)	22.61 ± 26.62
Duration, months (mean ± SD)	5.65 ± 4.54

Treatment outcomes

**Figure 2:**
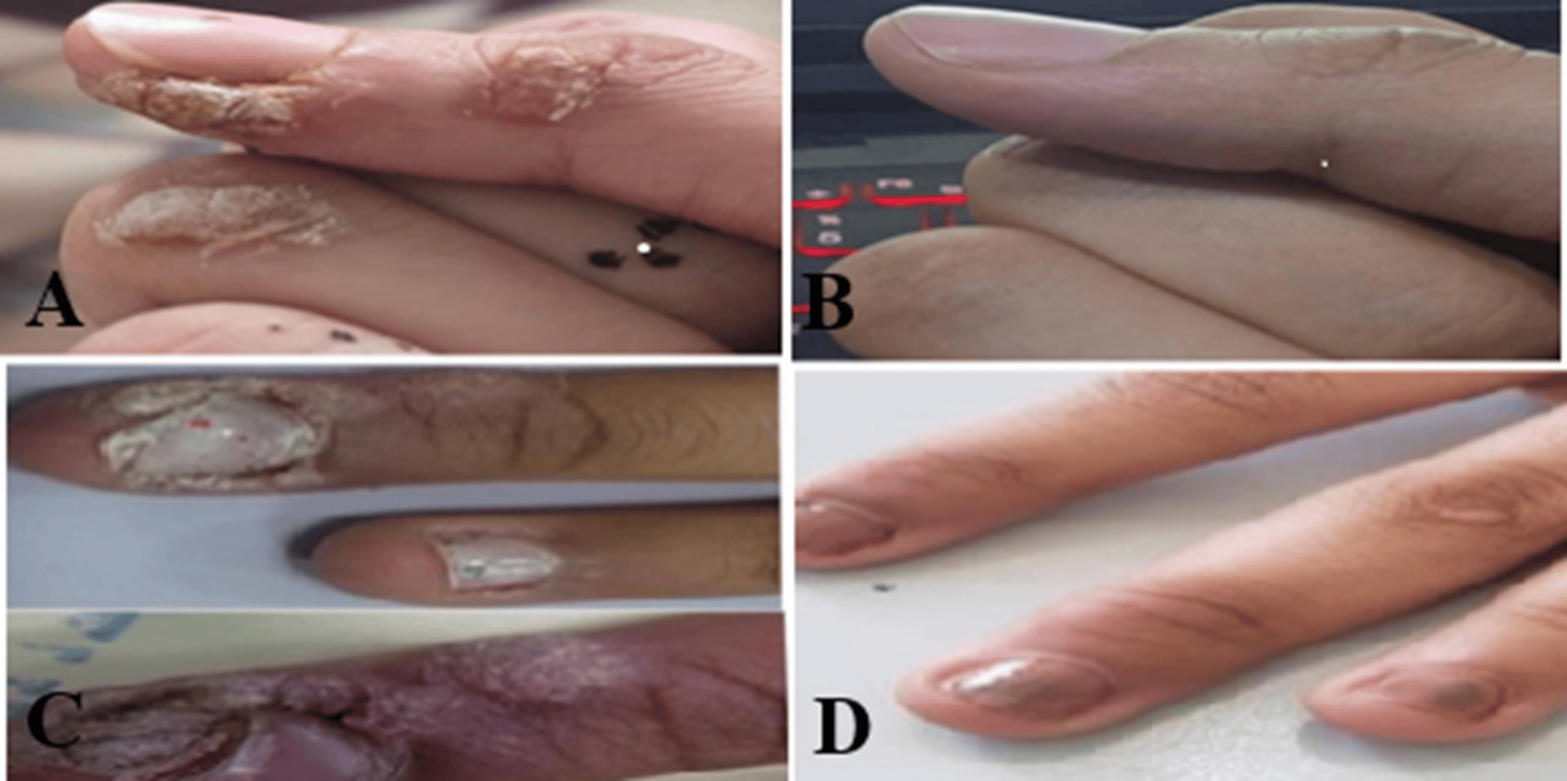
Representative clinical response to intralesional vitamin D3 injection in two patients. (A and C) Pre-treatment presentation of recalcitrant periungual and subungual verruca vulgaris (left panel). (B and D) Post-treatment presentation demonstrating complete clinical clearance and restoration of normal skin architecture following the final injection session (right panel). Source: Primary clinical data collected by the authors.

Complete clinical response was achieved in 36 patients (59.0%; 95% CI: 46.4-70.8). Partial response was observed in 20 patients (32.8%; 95% CI: 21.8-45.4), while five patients (8.2%; 95% CI: 2.7-18.1) showed no clinically meaningful improvement. The overall response rate was 91.8% (56/61 patients; 95% CI: 82.4-96.5) (Figure 2).

**Figure 3:**
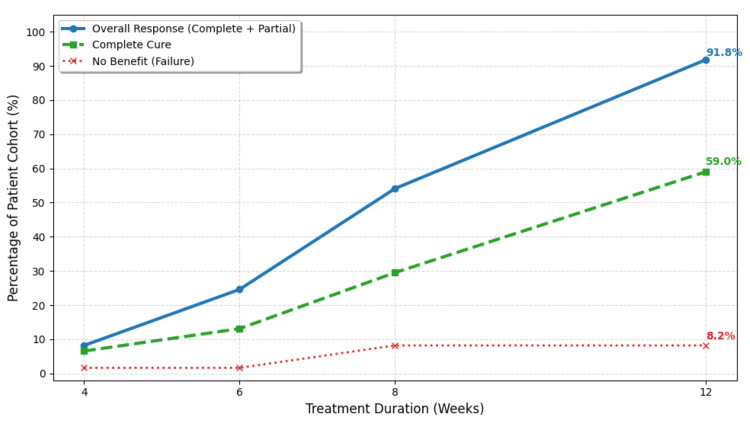
Cumulative clinical response and treatment failure over 12 weeks. The graph illustrates the clinical outcomes of 61 patients over six bi-weekly treatment sessions. The Overall Response (solid blue line) represents the cumulative percentage of patients with complete or partial improvement. The Complete Cure (dashed green line) indicates the rate of total clearance. The No Benefit (dotted red line) represents the 8.2% of patients who did not achieve a clinical response (≥50% reduction) by the final session. Source: Primary clinical data collected by the authors.

The speed of clinical improvement was progressive; while only 8.2% (n = 5) of patients showed an overall response after two sessions (four weeks), this increased significantly to 91.8% (n = 56) by the sixth session (Figure 3). Among the 36 patients (59.0%) who achieved complete cure, all completed six months of follow-up (100%).

Adverse events and safety

Injection-site pain was reported in 46 patients (75.4%). Transient localized erythema and swelling occurred in eight patients (13.1%). No cases of scarring, dyspigmentation, secondary infection, systemic toxicity, or treatment discontinuation were observed. No serious adverse events were reported. Safety outcomes are summarized in Table 2.

**Table 2:** Summary of adverse events and treatment tolerability (N = 61). Legend: Safety profile of intralesional vitamin D3 injections. Abbreviation: n, number of patients.

Adverse Event	n (%)
Procedural pain (during injection)	46 (75.4)
Localized swelling or erythema	8 (13.1)
Scarring	0 (0.0)
Secondary infection	0 (0.0)
Systemic toxicity	0 (0.0)
Treatment discontinuation	0 (0.0)

Recurrence

Among the 36 patients who achieved complete clinical response, no recurrence was observed during the six-month follow-up period. All patients achieving complete response completed the scheduled follow-up assessments.

Factors associated with complete response

Univariate logistic regression analysis showed that younger age was significantly associated with complete clinical response (OR: 0.87 per one-year increase, 95% CI: 0.81-0.93; p < 0.001). The mean age was 17.17 ± 6.82 years in patients with complete response compared with 32.28 ± 12.78 years in those without complete response (p < 0.001; Cohen’s d = -1.56).

Age-group analysis (≤20 years vs >20 years) demonstrated consistent findings, with complete response achieved in 32 of 43 patients (74.4%) aged ≤20 years compared with 4 of 18 patients (22.2%) aged >20 years (chi-square test, p < 0.001).

No statistically significant associations were identified between complete response and sex, wart subtype, lesion burden, prior treatment history, or lesion surface area (Table 3).

**Table 3:** Univariate logistic regression analysis of factors associated with complete cure (N = 61). Legend: Univariate predictors of treatment success. ORs and 95% CIs were calculated using unadjusted logistic regression. † p‑values < 0.05 are considered statistically significant. ‡ Odds ratio not calculated for plane warts due to zero events in the complete cure group. Abbreviations: OR, odds ratio; CI, confidence interval; SD, standard deviation; N/A, not applicable.

Variable	Complete Cure (n = 36)	Non‑complete Cure (n = 25)	OR (95% CI)	p‑value†
Age, years (mean ± SD)	17.17 ± 6.82	32.28 ± 12.78	0.87 (0.81-0.93)	<0.001
Age group, n (%)				
11-20 years	32 (88.9)	7 (28.0)	20.57 (5.29-79.94)	<0.001
Other ages	4 (11.1)	18 (72.0)	1 (Reference)	-
Sex, n (%)				
Female	27 (75.0)	20 (80.0)	0.75 (0.22-2.58)	0.648
Male	9 (25.0)	5 (20.0)	1 (Reference)	-
Wart type, n (%)				
Common	18 (50.0)	13 (52.0)	1 (Reference)	-
Plantar	11 (30.6)	7 (28.0)	1.13 (0.34-3.76)	0.834
Periungual	5 (13.9)	2 (8.0)	1.81 (0.29-11.18)	0.517
Mixed	2 (5.6)	2 (8.0)	0.72 (0.09-5.81)	0.760
Plane	0 (0.0)	1 (4.0)	N/A ‡	0.999
Prior treatment, n (%)				
Previously treated	19 (52.8)	11 (44.0)	1.42 (0.51-3.97)	0.501
Treatment‑naïve	17 (47.2)	14 (56.0)	1 (Reference)	-
Number of warts (mean)	1.92	2.00	0.94 (0.61-1.45)	0.786
Surface area, mm² (mean)	26.22	17.37	1.02 (0.99-1.04)	0.218

## Discussion

This study evaluated the association between intralesional vitamin D3 and clinical outcomes in patients with cutaneous warts. The complete response rate was 59.0%, with an overall response rate of 91.8%. No serious adverse events were recorded. These findings indicate that intralesional vitamin D3 was associated with favorable clinical outcomes and a favorable short-term safety profile in this cohort. However, because of the retrospective uncontrolled design, these findings should not be interpreted as evidence of treatment efficacy, as spontaneous wart regression, selection bias, and residual confounding cannot be excluded.

The complete response rate observed in the present study falls within the range reported for intralesional vitamin D3 in previous studies. Published complete response rates have varied substantially, ranging from approximately 40% in placebo-controlled trials to approximately 80% in selected uncontrolled cohorts, reflecting differences in wart subtype, lesion burden, disease chronicity, treatment protocols, patient selection, and study design [2,8-13]. Higher response rates reported in uncontrolled studies should be interpreted cautiously because spontaneous wart regression, selection bias, and outcome assessment bias may contribute to overestimation of treatment effects in the absence of a comparator [2,11].

In contrast, randomized controlled trials have demonstrated less consistent efficacy. Merry et al. reported no significant difference between intralesional vitamin D3 and placebo at 24 weeks (30% vs 31%), while Lahoria et al. found comparable clearance rates across vitamin D3 (66%), MMR (58%), MIP (55%), and placebo (64%) groups [11,14]. The discrepancy between randomized and observational studies suggests that the higher response rates reported in uncontrolled cohorts may partly reflect spontaneous regression, patient selection, and outcome assessment bias rather than treatment effects alone [11].

Current comparative evidence suggests that intralesional vitamin D3 has efficacy broadly comparable to other intralesional immunotherapies rather than demonstrating consistent superiority [5,15-17]. Reported complete response rates vary across agents, including *Candida* antigen (60%-76.7%), MMR (58%-86.7%), and vitamin D3 (48%-76.7%) [15-17]. Overall, available data suggest that vitamin D3 is one of several comparable intralesional immunotherapy options rather than a superior modality. Its clinical utility may therefore relate to low cost, accessibility, and favorable tolerability, particularly in resource-limited settings.

Treatment protocols differed considerably across published studies, with doses ranging from 12,000 to 300,000 IU per session, injection intervals of two to four weeks, and treatment durations of two to seven sessions [2,8-11,14,18]. Most studies, including the present cohort, employed repeated biweekly injections and reported progressive clinical improvement over successive treatment sessions. Although differences in dose intensity and treatment frequency may contribute to heterogeneous outcomes, direct comparisons are limited by variations in wart subtype, disease severity, patient selection, and study design.

Notably, Merry et al. used a substantially lower dose of intralesional vitamin D3 (12,000 IU) every four weeks for up to three sessions and found no benefit over placebo, with complete clearance rates of 30% versus 31% at 24 weeks [11]. By contrast, Lahoria et al. administered injections every two weeks for up to seven sessions and reported complete clearance in 66% of injected warts, with a mean time to complete clearance of 7.4 weeks [14].

Vitamin D3 is hypothesized to exert its therapeutic effects through immunomodulatory mechanisms, including enhanced keratinocyte differentiation and induction of antimicrobial peptides such as cathelicidin and β-defensins [4,6,19]. Experimental studies demonstrating increased cathelicidin expression following intralesional vitamin D3 administration provide biological plausibility for this mechanism [20]. Occasional clearance of untreated lesions has also been reported, suggesting localized immune activation, although systemic immunologic effects have not been conclusively demonstrated [8,9].

No recurrence was observed among complete responders during six months of follow-up; however, the precision of this estimate is limited by the modest sample size and relatively short follow-up period, and prior studies have reported variable recurrence rates following intralesional vitamin D3 therapy [9,10,20]. A systematic review and meta-analysis suggested favorable recurrence profiles with vitamin D3 compared with several alternative intralesional immunotherapies and placebo [5].

Some clinical studies have reported lower serum vitamin D levels in patients with cutaneous warts, although findings remain inconsistent [21,22]; however, baseline vitamin D status has not been shown to correlate with treatment response, supporting a local rather than systemic mechanism.

Adverse events were mild and limited to local reactions, primarily injection-site pain and transient erythema or swelling. No scarring, dyspigmentation, or systemic toxicity was observed. The absence of systemic adverse events in the present cohort is consistent with previous reports evaluating intralesional vitamin D3 [8-10,23].

Younger age was associated with a higher likelihood of complete response, consistent with prior studies reporting better outcomes with intralesional immunotherapy in younger patients [15,24]. One possible explanation is age-related differences in immune responsiveness or disease chronicity; however, this hypothesis requires confirmation in prospective studies. No statistically significant associations were identified between response and wart type, lesion burden, or prior treatment history.

Although cutaneous warts generally affect males and females with similar frequency, female patients predominated in the present cohort [25]. This imbalance may reflect referral patterns, healthcare utilization, or sampling variability inherent to the retrospective single-center design rather than the underlying epidemiology of the disease. Accordingly, caution is warranted when generalizing these findings to other populations.

Study limitations

This study has several limitations. The retrospective uncontrolled design limits causal inference and introduces potential selection bias and confounding. The sample size was modest and derived from two centers in a single geographic region. Standardized severity scoring was not used, and outcome assessment was not blinded, introducing potential observer bias. HPV genotyping, immunologic markers, and serum vitamin D levels were not assessed. The follow-up period of six months may have been insufficient to detect late recurrence.

Future randomized controlled trials with standardized protocols, larger sample sizes, head-to-head comparisons with established immunotherapies, and longer follow-up are required to define the comparative efficacy and long-term role of intralesional vitamin D3 in cutaneous warts.

## Conclusions

Intralesional vitamin D3 was associated with a complete response in approximately 60% of patients and demonstrated a favorable short-term safety profile in this retrospective cohort. Further adequately powered randomized controlled trials with standardized treatment protocols and longer follow-up are required to determine the comparative effectiveness and long-term safety of intralesional vitamin D3.
